# Acacetin alleviates myocardial ischaemia/reperfusion injury by inhibiting oxidative stress and apoptosis via the Nrf-2/HO-1 pathway

**DOI:** 10.1080/13880209.2022.2041675

**Published:** 2022-03-04

**Authors:** Chan Wu, Ruo-Lan Chen, Yan Wang, Wei-Yin Wu, Gang Li

**Affiliations:** Institute of Cardiovascular Research, Xiamen Cardiovascular Hospital of Xiamen University, School of Medicine, Xiamen University, Xiamen, Fujian, China

**Keywords:** Ventricular premature, ventricular tachycardia, ventricular fibrillation, SOD1, collagen, Bax, Bcl-2, TLR4, IL-6, inflammation

## Abstract

**Context:**

Acacetin is a natural source of flavonoids with anti-inflammatory and antioxidant effects.

**Objective:**

This study determines acacetin’s protective effect and mechanism on myocardial ischaemia/reperfusion (I/R) injury.

**Materials and methods:**

Sprague-Dawley rats were divided into sham and I/R injury and treatment with acacetin. Acacetin (10 mg/kg) was subcutaneously injected for 7 days. ECG and echocardiography were conducted to determine arrhythmia and heart function. The pathological characters of the heart were determined with triphenyl tetrazolium chloride staining, Haematoxylin & Eosin staining, and Masson staining. Expression of proteins in infarct tissues was examined with western blots.

**Results:**

Administrated with acacetin in I/R rats significantly reduced the arrhythmia score from 4.90 to 2.50 and the reperfusion arrhythmia score from 3.79 to 1.82 in the vehicle or the acacetin group, respectively. LVEF was improved from 33.5% in the I/R group to 43.7% in the acacetin group, LVFS was increased from 16.4% to 24.5%, LVIDs was decreased from 6.5 to 5.3 mm. The inflammatory cell infiltration, myocardial fibrosis, and collagen 1 and 3 were reduced by acacetin. Acacetin promoted SOD and decreased MDA. In myocardial tissues, the expression level of TLR4 and IL-6 were restrained, and IL-10 was promoted. Apoptotic protein Bax was suppressed, and anti-apoptotic protein Bcl-2 was promoted in the acacetin group. Interestingly, the transcription factor Nrf-2/HO-1 pathway was also reversed by acacetin.

**Discussion and conclusion:**

Our findings indicated that acacetin has a potential therapeutic effect in clinical application on treating I/R-induced heart injury.

## Introduction

Ischaemic heart disease is the deadliest disease globally (Budas et al. 2010). Through thrombolysis or interventional therapy, the myocardium in the ischaemic area is reperfused, and the mortality rate of acute myocardial infarction is significantly reduced (Hausenloy et al. 2019). However, although reperfusion improves myocardial blood supply, myocardial damage is caused by ischaemia, leading to arrhythmia, increased infarct size, and persistent ventricular dysfunction, called myocardial ischaemia/reperfusion injury (I/R; Rassaf et al. 2014). Myocardial I/R is the leading cause of ventricular remodelling in patients with myocardial infarction and eventually progressing to heart failure (Azevedo et al. 2016). Previous findings have demonstrated that the main mechanisms of myocardial I/R involve oxidative stress, inflammation, mitochondrial damage, apoptosis, autophagy, intracellular calcium overload, and abnormal energy metabolism (Heusch et al. 2011; Wei et al. 2018). Myocardial I/R-induced oxidative stress is the primary factor for cardiomyocyte apoptosis (Ghafouri-Fard et al. 2020a). Various factors are interrelated, and there is still much work to be done. Therefore, in-depth exploration of the mechanism of myocardial I/R can provide an essential experimental basis for effectively controlling the mortality of patients with myocardial infarction and the incidence of heart failure.

Acacetin was initially isolated from the traditional Chinese medicinal material Tianshan Saussurea, and it is widely found in many plants, such as the *Acacia* tree, fragrant *Eupatorium* herb, and *Chrysanthemum* (Chang et al. 2017). Acacetin is a natural flavonoid compound with a potent antioxidant capacity similar to other flavonoid compounds (Li et al. 2008). Studies have shown that acacetin has a significant inhibitory effect on potassium channels and can effectively treat atrial fibrillation and arrhythmia (Li et al. 2008; Wu et al. 2011). Our team has focussed on the effect of acacetin on potassium channels and determined that the anti-atrial fibrillation effect of acacetin was based on small conductance Ca^2+^-activated potassium currents (Chen et al. 2017). Acacetin has multiple biological activities, such as antioxidation, anti-inflammatory, anti-bacterial, anti-osteoporosis, anti-tumor, immune regulation, and heart protection (Ni et al. 2017; Wu et al. 2018, 2020). The pathogenesis of I/R is more complicated, so acacetin may potentially treat I/R.

Our team has proved that acacetin can improve doxorubicin induced-cardiomyopathy and hypoxia/reoxygenation (HR) injury of H9C2 cardiomyoblasts and primary cultured neonatal rats cardiomyocytes via the AMPK/Nrf2 signalling pathway (Wu et al. 2018, 2020). This study used a model of ischaemia for 35 min and reperfusion for 7 days to investigate the effect of therapeutic administration of acacetin and the specific mechanism.

## Materials and methods

### Experimental materials and animals

Acacetin in this study was synthesized as previously described (Chen et al. 2017; Wu et al. 2018). Sprague-Dawley (SD) rats (male, 6–8 weeks) used in the experiment were purchased from Shanghai SLAC Laboratory Animal Ltd. All animal experiment procedures were approved by the Ethics Committee of Xiamen University.

### Rat I/R model and drug administration

As previously described, the rat I/R injury was introduced (Zhang et al. 2010; Wei et al. 2018). A total of 36 rats were divided into 4 groups: sham group (*n* = 10), sham rats treated with acacetin group (*n* = 6), vehicle group (I/R rats injected with saline; *n* = 9), and I/R rats treated with acacetin group (n = 11; subcutaneously injected with 10 mg/kg acacetin on the neck of rats 15 min before the I/R injury conducted; Liu H et al. 2016; Tian et al. 2019; Liu et al. 2021). After 35 min of ischaemia, rats were reperfused. Rats were subcutaneously injected with acacetin (10 mg/kg) or saline (equal volume of normal saline) on the neck of rats twice a day for 7 days.

### Echocardiography

After 7 days of treatment on the rats, echocardiography was conducted using Vevo 2100 (VisualSonics Inc, Toronto, Canada) to detect cardiac function. Four parameters, left ventricular ejection fraction (LVEF), left ventricular fraction shortening (LVFS), left ventricular internal diameter end-diastole (LVIDd), and end-systole (LVIDd), were measured at least three consecutive cardiac cycles (Fan et al. 2013; Lin et al. 2015).

### Triphenyl tetrazolium chloride (TTC) staining

After the rats were sacrificed, the heart tissues were cut into 1–2 mm slices perpendicular to the long axis of the heart. The pieces were then placed in 2% TTC staining solution preheated at 37 °C for 10 min. After staining, the slices were placed on a colorimetric plate with a scale and take a picture (Wei et al. 2018; Yang et al. 2018). Image J software was used to calculate the infarct size.

### Haematoxylin & eosin (HE) staining

The ventricular tissues were embedded with OCT and sectioned into 6 μm slices. Sections were fixed in 4% paraformaldehyde and stained with eosin, and haematoxylin was used for nuclei staining as previously described (Hou et al. 2019; Wei et al. 2019).

### Masson staining

After being fixed, the sections were stained with haematoxylin for the nucleus. The cytoplasm was stained with ponceau staining solution and finally staining with phosphomolybdic acid aqueous solution and aniline blue staining solution. After sealing with neutral resin, pictures were captured with a microscope (Xing et al. 2020; Yu et al. 2020).

### SOD and MDA level detection in serum

Serum was obtained by centrifuge at 4000 rpm for 10 min. The SOD and MDA kit (Beyotime Biotechnology, Shanghai, China) were employed to detect the serum level of SOD and MDA following the manufacturer’s instructions.

### IL-6 Level detection in serum

The serum level of IL-6 was conducted using an IL-6 ELISA kit (Cat No: PI328, Beyotime Biotechnology, Shanghai, China) following the manufacturer’s instructions.

### Western blot

After minced the rat heart samples in RIPA lysis buffer, the samples were supplemented with loading buffer and denatured at 95 °C for 5–10 min. The protein was separated with SDS-PAGE gel and transferred to the PVDF membrane. After being blocked with 5% skim milk for 1 h, the specific primary antibody was incubated overnight at 4 °C, and the secondary antibodies were incubated for an additional 1 h. Then the membranes were developed in the FluorChem E chemiluminescence imaging system. Image J image processing software performed band analysis. The primary antibodies used in the experiment were partly bought from Santa Cruz Biotechnology, including anti-IL-10 (sc-365858), anti-Bcl-2 (sc-7382), anti-Bax (sc-493), anti-TLR-4 (sc-293072), anti-GAPDH(A-3; sc-137179), or from Wuhan Boster Biological Technology, including anti-Nrf2 (PB0327), anti-HO-1 (PB0212), and anti-SOD1 (BA1401). The primary antibodies, such as anti-collagen I (67288), anti-collagen III (22734), and the secondary antibodies, such as peroxidase affiniPure goat anti-mouse IgG (H + L), peroxidase AffiniPure Goat Anti-Rabbit IgG (H + L), were from Proteintech Technology.

### Statistical analysis

GraphPad Prism 8 statistical software was used for data analysis and graphing. Measurement data were expressed as Mean ± SEM. Differences between multiple groups were calculated by one-way ANOVA followed by Tukey’s *post ho*c test. The incidence of arrhythmia Fisher’s test was used for statistics, and *P* < 0.05 was considered statistically significant.

## Results

### Effects of acacetin on cardiac infarction and arrhythmia in rats

**Figure 1.**
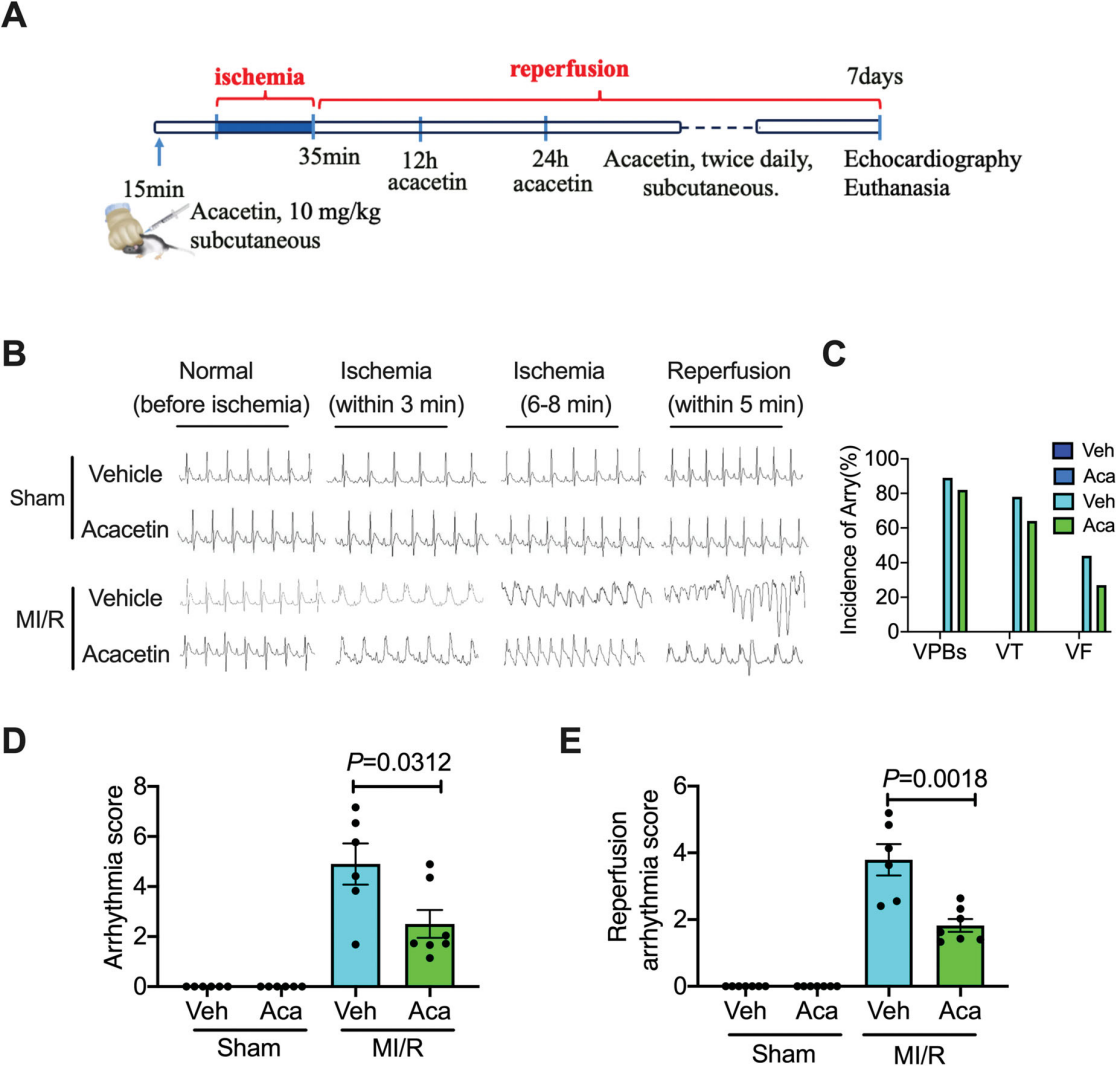
Acacetin alleviated cardiac arrhythmia induced by ischaemia/reperfusion. (A) Graphic image of myocardial I/R injury induction and acacetin administration. (B) The typical electrocardiograms were recorded before ischaemia, 1 min after ischaemia, 6–8 min after ischaemia, and 5 min after reperfusion. Quantitative of arrhythmia score (C), reperfusion arrhythmia (D), and incidence of arrhythmia (E).

In this study, the ECG of the rat was detected during ischaemia. The results in Figure 1(A) showed that ligation of the left anterior descending coronary artery cause ST-segment elevation indicated the success of the myocardial ischaemia model. Acacetin treatment did not influence the ECG of the sham rats. After ischaemic 6 min, the ECG tends to cause arrhythmia, aggravated after reperfusion in the I/R treated with vehicle. However, the administration of acacetin in I/R rats promoted the recovery of rats’ ECG to a normal rhythm. It reduced the possibility of arrhythmia, including ventricular premature (VPBs), ventricular tachycardia (VT), and ventricular fibrillation (VF) during ischaemia and reperfusion (Figure 1(B–D)).

### Effect of acacetin on left ventricular dysfunction, inflammation infiltration, and fibrosis induced by I/R in rats

TTC staining was performed to detect rat myocardial infarcted area 7 days after reperfusion. The results showed treatment with acacetin did not exhibit any difference compared to sham rats. However, the infarct area of the I/R rats treated with vehicle reached about 30%. The I/R rats administrated with acacetin reduced the infarcted size to less than 20%.

**Figure 2.**
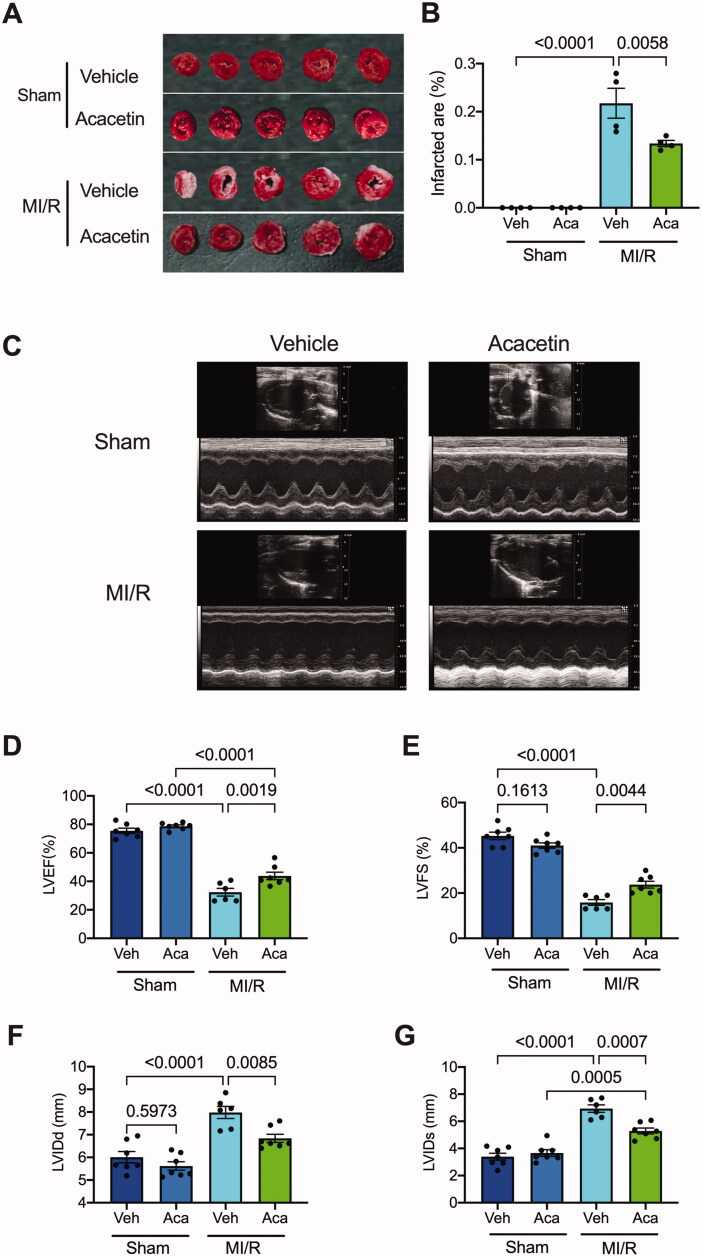
Acacetin reduced ischaemic area and attenuated cardiac function in ischaemia/reperfusion rats. (A) TTC staining of rat hearts 1 week after I/R. (B) Quantitative statistics of left ventricular myocardial infarction area (*n* = 4). (C) Representative echocardiography in four groups. Left ventricular ejection fraction (LVEF) (D), fractional shorting (LVFS) (E), left ventricular internal diameter at end-diastole (LVIDd) (F), and left ventricular internal diameter at end-systole (LVIDs) (G) were analysed.

Echocardiography was employed to determine the left ventricular function of rats. As shown in Figure 2(A–B), treatment with acacetin did not affect the myocardial function in sham rats. After I/R in rats, LVEF (Figure 2(D)) and LVFS (Figure 2(E)) were significantly reduced compared with the sham group, while the two values in the acacetin group were restored considerably. Besides, I/R caused the increase of left ventricular internal diameter at end-diastole (LVIDd; Figure 2(F)) and left ventricular internal diameter at end-systole (LVIDs; Figure 2(G)) in the vehicle group compared to the sham group, and acacetin reversed the level of LVIDs. The level of LVIDd was slightly reduced by acacetin without a significant difference. These results indicated that acacetin significantly improved left ventricular function induced by I/R.

**Figure 3.**
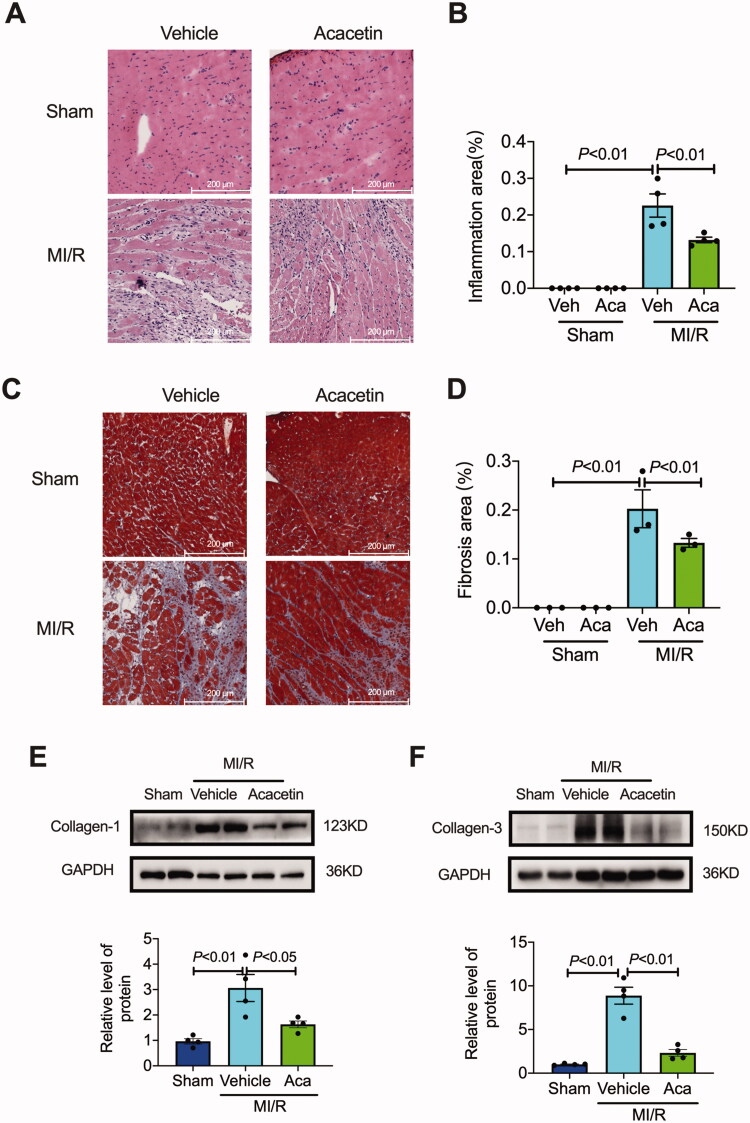
Acacetin reduced myocardial inflammation and fibrosis in rats. (A,B) Representative images and quantitative analysis (*n* = 4) of haematoxylin-eosin (H&E) staining were obtained 1 week after I/R (Scale bar indicated 200 μm). (C,D) Representative images and quantitative analysis (*n* = 4) of Masson staining were detected 1 week after I/R (Scale bar indicated 200 μm). (E,F) Representative western blot image and quantitative analysis of collagen-1 and collagen-3 in rat hearts (*n* = 4).

H&E staining and Masson staining were applied to evaluate the structural damage and fibrosis of the rat heart (Wei et al. 2019; Xing et al. 2020). The results of H&E staining indicated that I/R caused inflammatory cell infiltration in myocardial tissue, and this structural change could be reduced by treatment with acacetin (Figure 3(A,B)). In the Masson staining, the myocardial tissue was red, and the fibrous tissue was blue-purple. There is not any difference between vehicle and acacetin-treated sham rats. Compared to the sham rats, the I/R rats significantly increased the area of fibrous tissue, and administration of acacetin can considerably reduce the fibrosis of myocardial tissue (Figure 3(C,D)). In addition, the expression level of collagen-1 (Figure 3(E)) and collagen-3 (Figure 3(F)) in myocardial tissue was significantly increased due to I/R, and this increasing trend can be inhibited by treatment with acacetin. In summary, acacetin can remarkably reduce the inflammatory cell infiltration and fibrosis in myocardial tissue of I/R rats.

### Effect of acacetin on oxidative stress, inflammation induced by I/R in rats

**Figure 4.**
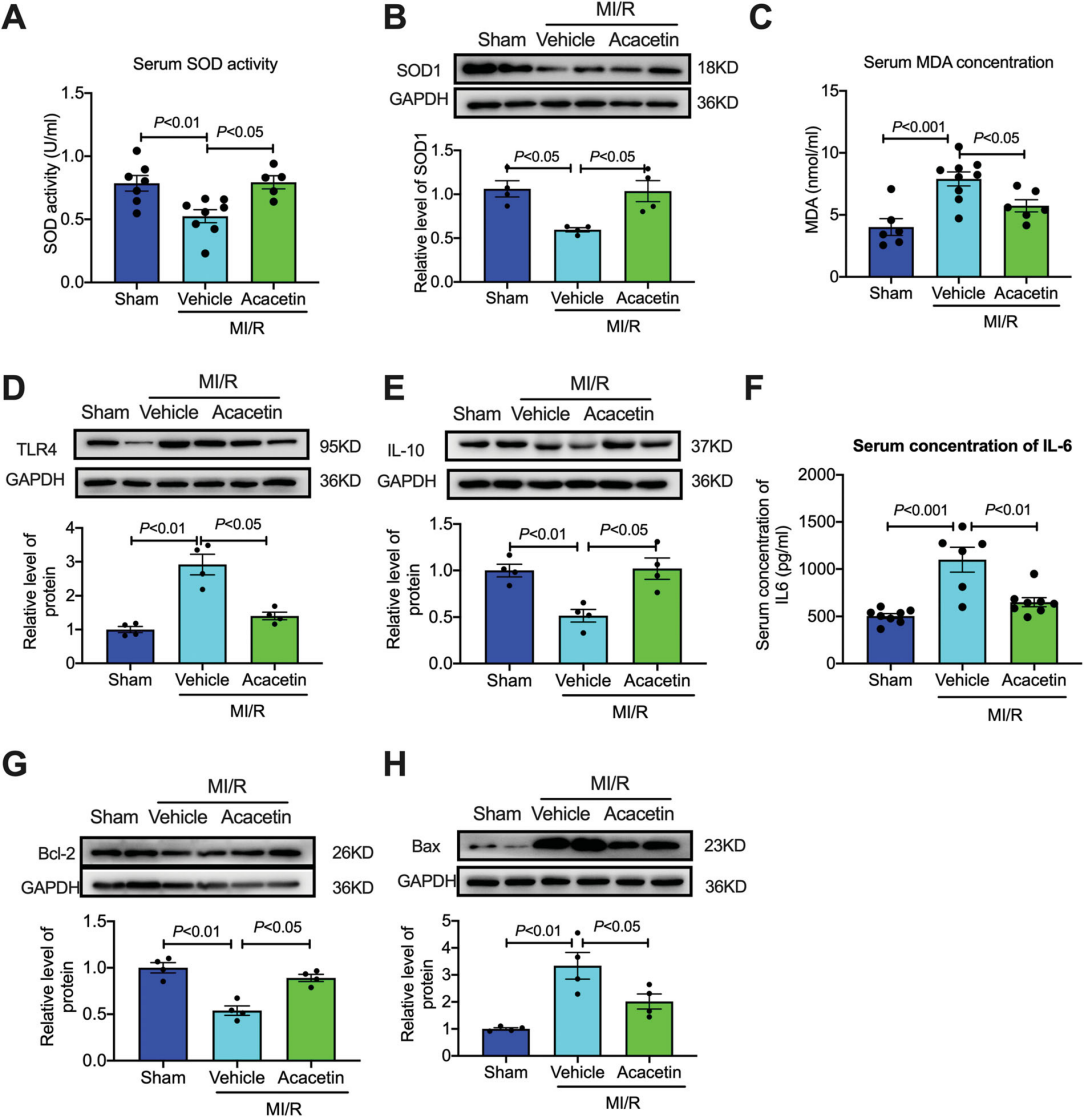
Acacetin inhibited oxidative stress, inflammation, and apoptosis after I/R. (A) The levels of SOD in rat serum were detected (*n* = 5–8). (B) Representative western blot and quantitative analysis of SOD-1 expression in rat heart tissues (*n* = 4). (C) The levels of MDA in rat serum (*n* = 5–8). (D) Representative western blot images and quantitative analysis of TLR4 expression in rat hearts (*n* = 4). (E) Representative western blot images and quantitative analysis of IL-10 expression in rat hearts (*n* = 4). (F) The levels of IL-6 in rat serum (*n* = 6–9). (G) Representative western blot images and quantitative analysis of Bcl-2 expression in rat hearts (*n* = 4). (H) Representative western blot images and quantitative analysis of Bax expression in rat hearts (*n* = 4).

The levels of superoxide dismutase (SOD) and malondialdehyde (MDA) reflect the level of oxidative stress (Chien et al. 2020). First, we measured SOD (Figure 4(A)) and MDA (Figure 4(C)) levels in the rat serum. The results showed that the SOD level in the vehicle group was decreased, and the MDA level was increased, and treatment with acacetin restored SOD levels and restrained the increase of MDA levels. Furthermore, we detected SOD-1 protein expression level (Figure 4(B)) in myocardial tissue, and the results also showed that acacetin significantly inhibited the decrease of SOD level caused by I/R.

Inflammatory factors, such as toll-like receptor 4 (TLR4), interleukin-6 (IL-6), and interleukin-10 (IL-10), play vital roles in the inflammatory reaction of cardiomyocytes. The expression levels of TLR4 in the vehicle group were significantly increased in the cardiac tissue, and the serum level of IL-6 and acacetin could dramatically inhibit the surge of TLR4 (Figure 4(D)) and IL-6 (Figure 4(F)). Moreover, the level of IL-10 also decreased due to I/R, and intervention of acacetin increased IL-10 levels (Figure 4(E)).

Cell apoptosis occurs after inflammation and oxidative stress in cardiac I/R. The results showed that the proapoptotic protein was increased, and anti-apoptotic protein Bcl-2 was decreased in the I/R group. At the same time, acacetin significantly inhibited the decrease of Bcl-2 expression (Figure 4(G)) and the increase of Bax (Figure 4(H)). These effects of acacetin also proved that acacetin exerted the cardioprotective influence by reducing oxidative stress, inflammation, and apoptosis.

### Effect of acacetin on Nrf-2/HO-1 pathway in rats

**Figure 5.**
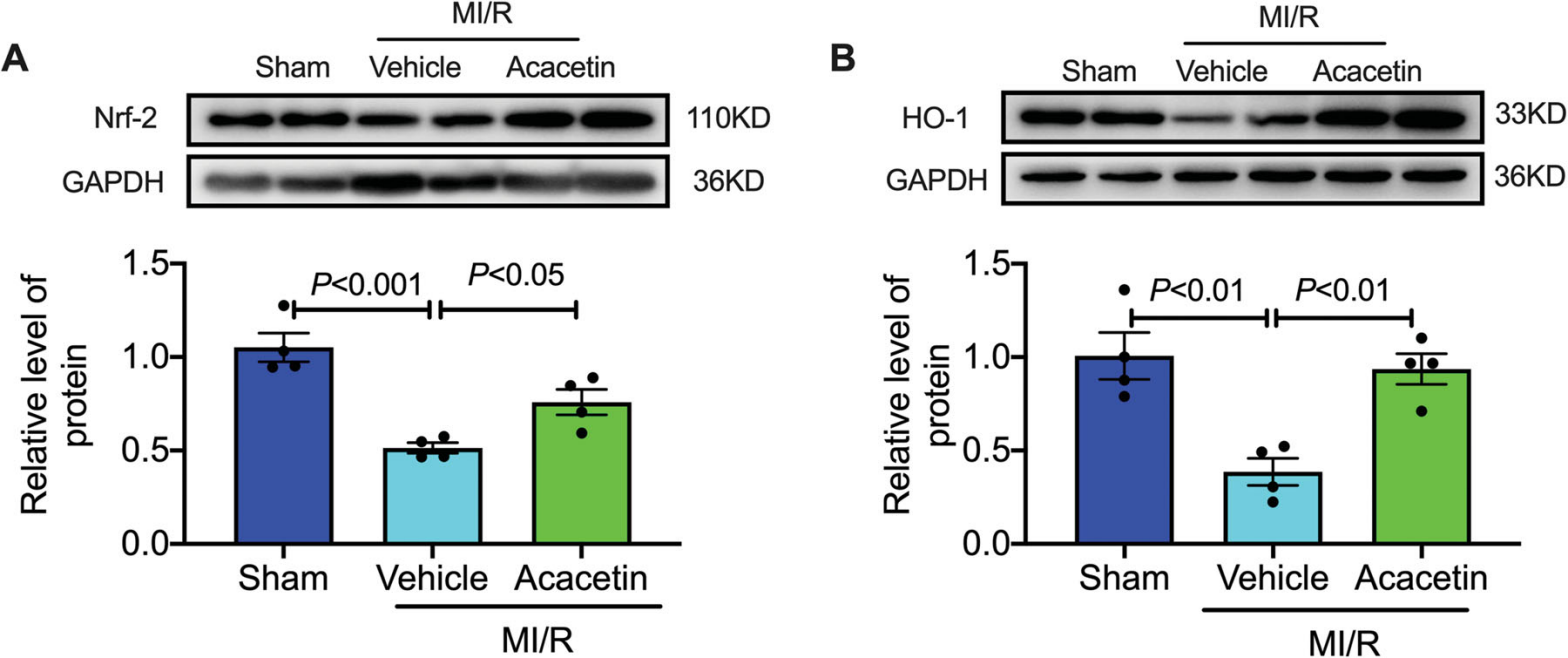
Acacetin regulated the Nrf-2/HO-1 signalling pathway after I/R. Representative western blot images and quantitative analysis of Nrf-2 (A) and HO-1 (B) expression in rat hearts (*n* = 4).

The Nrf-2/HO-1 pathway is an essential regulator in the process of I/R. The results showed that the vehicle group significantly reduced the expression of Nrf-2 (Figure 5(A)) and HO-1 (Figure 5(B)) in myocardial tissue compared to the sham group. After administration of acacetin, Nrf-2, and HO-1 in myocardial tissue was significantly restored. These results indicate that acacetin can protect the I/R cardiac by regulating the Nrf-2/HO-1 signalling pathway.

## Discussion

Acacetin is an *O*-methylated flavone extracted from the traditional Chinese medicine Xuelianhua or many other plants as a bioactive compound (Chen et al. 2017). In the present study, a rat model for myocardial ischaemia/reperfusion injury (I/R) was conducted, and acacetin was administrated to the rats for 7 days before or after reperfusion. Our results showed that acacetin treatment reduced the incidence of arrhythmia and the area of myocardial infarction. The echocardiography and H&E staining proved that acacetin improved cardiac function and reduced the structural damage of myocardial tissue. Myocardial I/R can lead to myocardial fibrosis. The normal myocardial interstitium is composed of cellular and non-cellular structures. Each group of collagen components together form a collagen fibre network, which affects the structure and function of the heart. Myocardial fibrosis is mainly manifested by increased deposition and disordered arrangement of collagen in the interstitium (Yu et al. 2020). In the results, myocardial I/R injury elevated inflammatory infiltration as shown in H&E staining and promoted myocardial fibrosis (Masson staining) by promoting the expression of Collagen 1 and Collagen 3. One week after administration, acacetin alleviates cardiac I/R injury, reducing inflammatory infiltration and myocardial fibrosis by decreasing Collagen 1 and Collagen 3, which indicated that acacetin plays a cardioprotective effect after I/R.

When myocardial I/R occurs, abnormal activation of the xanthine oxidase system and neutrophils and abnormal mitochondrial respiratory function produce excessive reactive oxygen species, which can cause oxidative stress and cell apoptosis, and damage to myocardial function (Monji et al. 2013; Xu et al. 2018). After the oxidative stress-induced, cell membrane lipids undergo peroxidation to generate a large amount of MDA, accompanied by the consumption of antioxidant enzyme SOD (Choudhary et al. 2008; Monji et al. 2013). MDA and SOD are often used as markers to measure oxidative stress *in vivo*. Acacetin is a flavonoid compound, which has the antioxidant capability. Our study also verified the effect of acacetin on myocardial I/R; that is, acacetin can significantly reduce the level of MDA and restore the antioxidant capacity of SOD. Therefore, acacetin can protect against myocardial I/R by inhibiting oxidative stress.

The inflammatory response is activated at the stage of myocardial ischaemia and is significantly increased during reperfusion (de Haan et al. 2017). The inflammatory response process includes chemotaxis, infiltration of inflammatory cells, and the synthesis and secretion of cytokines during myocardial I/R (Wu et al. 2020). TLR4 is a transmembrane protein regarded as a pattern recognition receptor (PRR). Its activation results in the induction of the NF-κB pathway and the production of inflammatory cytokines, contributing to the innate immune system (Ghafouri-Fard et al. 2021). As a critical inflammatory regulator, TLR4 can activate NF-κB and IRF3 through the MyD88-dependent pathway and the TRIF-dependent pathway in I/R cardiac, stimulating the expression of pro-inflammatory and immunoregulatory cytokine genes and mediating cascade inflammation (Ramalingam et al. 2019). In this study, acacetin significantly reduced the increase of TLR4 expression caused by I/R, indicating that acacetin can inhibit TLR4-mediated signalling pathways and play an anti-inflammation effect. In addition, other cytokines, IL-6 and IL-10, play an essential role in the inflammatory response in the I/R process (Halade et al. 2013). During I/R, the cytokine IL-6 and IL-10 will be abnormally expressed (Wu et al. 2020). IL-6 and IL-10 are at the pivotal position of inflammation regulation, affecting neutrophils to flow into ischaemic myocardial tissue and damage myocardial structure and function (Ramalingam et al. 2019). Our study verified that myocardial I/R could increase the IL-6 level and decrease the IL-10 level, while acacetin restrained the increase of IL-6 and promoted the IL-10 level.

Acacetin can reduce the inflammatory response of myocardial tissue by inhibiting the levels of TLR4 and IL-6 and increasing the levels of IL-10. Apoptosis in myocardial tissue results from multiple factors, such as myocardial I/R-induced oxidative stress and inflammatory (Wang et al. 2020). In this study, acacetin significantly reduced the expression of the pro-apoptotic protein Bax and promoted the anti-apoptotic protein Bcl-2 level. Therefore, we proved that acacetin could inhibit oxidative stress and inflammation, inhibiting myocardial cell apoptosis and protecting the heart.

Nrf2 is an important transcription factor regulating cell protection mechanisms such as antioxidant and anti-inflammatory (Liu et al. 2019). When ischaemia and hypoxia, a large number of inflammatory factors and oxygen free radicals are produced; at this time, Nrf2 in the cytoplasm is dissociated from its cytoplasmic binding protein, and then transported across the nuclear membrane, enters the nucleus, and interacts with Nrf2-antioxidant response element (ARE; Wang et al. 2016; Yu et al. 2018). It initiates the expression of HO-1, thereby exerting anti-inflammatory, antioxidant, and anti-apoptotic effects in various diseases. HO-1 and its catalytic products carbon monoxide, iron, and bilirubin are also critical endogenous protective systems of the body (Zhang et al. 2020). They play a protective role in tissues and organs through multiple mechanisms such as antioxidation, anti-inflammatory, and anti-apoptosis (Liu et al. 2019). Studies have shown that the Nrf2/HO-1 signalling pathway plays an important protective role on myocardial I/R, and Nrf2 plays a myocardial protective role by regulating cardiomyocyte apoptosis, antioxidation, and inflammation (Zhou et al. 2014; Zhang et al. 2020). A series of studies demonstrated different microRNAs (miRNAs), such as miR-24-3p, miR-93, and miR-153, related I/R injury-induced cardiomyocytes apoptosis by regulating the Nrf-2/HO-1 pathway (Tang et al. 2018; Xiao et al. 2018; Ghafouri-Fard et al. 2020b; Hou et al. 2020). In this study, myocardial I/R caused a decrease in the expression of Nrf2 and HO-1. Acacetin administration promoted the body to initiate the endogenous defense mechanism by activating nuclear factor Nrf2 and up-regulating HO-1 expression to exert myocardial protection, which indicated the Nrf2/HO-1 pathway is the main regulating pathway for the protective effect of acacetin.

## Conclusions

Our present study demonstrated a novel protective effect of acacetin against myocardial I/R injury by reducing arrhythmia, myocardial infarction area and fibrosis, and restoring functional cardiac damage and structural damage. The protective function of acacetin on the I/R heart is mainly through regulating the Nrf2/HO-1 signalling pathway, thereby reducing oxidative stress and inflammation and inhibiting cell apoptosis.
